# *In silico* MCMV Silencing Concludes Potential Host-Derived miRNAs in Maize

**DOI:** 10.3389/fpls.2017.00372

**Published:** 2017-03-28

**Authors:** Muhammad Shahzad Iqbal, Basit Jabbar, Muhammad Nauman Sharif, Qurban Ali, Tayyab Husnain, Idrees A. Nasir

**Affiliations:** ^1^Center of Excellence in Molecular Biology, University of the PunjabLahore, Pakistan; ^2^Institute of Biochemistry and Biotechnology, University of the PunjabLahore, Pakistan

**Keywords:** maize chlorotic mottle virus (MCMV), miRanda, TapirHybrid, Targetfinder, RNA22, R language, miRNA, target prediction

## Abstract

Maize Chlorotic Mottle Virus (MCMV) is a deleterious pathogen which causes Maize Lethal Necrosis Disease (MLND) that results in substantial yield loss of Maize crop worldwide. The positive-sense RNA genome of MCMV (4.4 kb) encodes six proteins: P32 (32 kDa protein), RNA dependent RNA polymerases (P50 and P111), P31 (31 kDa protein), P7 (7 kDa protein), coat protein (25 kDa). P31, P7 and coat protein are encoded from sgRNA1, located at the 3′end of the genome and sgRNA2 is located at the extremity of the 3′genome end. The objective of this study is to locate the possible attachment sites of *Zea mays* derived miRNAs in the genome of MCMV using four diverse miRNA target prediction algorithms. In total, 321 mature miRNAs were retrieved from miRBase (miRNA database) and were tested for hybridization of MCMV genome. These algorithms considered the parameters of seed pairing, minimum free energy, target site accessibility, multiple target sites, pattern recognition and folding energy for attachment. Out of 321 miRNAs only 10 maize miRNAs are predicted for silencing of MCMV genome. The results of this study can hence act as the first step towards the development of MCMV resistant transgenic Maize plants through expression of the selected miRNAs.

## Introduction

Maize Chlorotic Mottle Virus (MCMV) (Tombusviridae: Machlomovirus) is one of the most harmful pathogen of maize (*Zea mays*) which, independently or synergistically with one or more of the viruses from other members of Potyviridae family such as Sugarcane Mosaic Virus (SCMV), causes significant yield losses in maize crop by causing Maize Lethal Necrosis Disease (MLND) (Bockelman et al., 1982; Wu et al., 2013). The disease is characterized by yellow streaks, chlorotic mottle and subsequent leaf damage by necrosis; these conditions can lead to functional abnormalities in plants and plant death (Nelson et al., 2011; Wangai et al., 2016). MCMV first emerged in Peru in 1974 (Castillo and Hebert, 1974) and afterwards in America (Uyemoto et al., 1980; Jiang et al., 1992) and has recently been detected in different regions around the world, including China (Xie et al., 2011), Taiwan (Deng et al., 2015), and Africa (Lukanda et al., 2016; Wangai et al., 2016).

MCMV is known to be transmitted by various means such as through insect vectors (beetles, mites, stem borers and aphids), infected soils and human activities (Kiruwa et al., 2016). Moreover, increasing international trade of maize seeds has been pointed out to incur a high risk of MCMV transmission between countries (Liu et al., 2016). Biological indexing, ELISA, electron microscopy, RT-PCR and surface plasmon resonance are among the frequently used detection approaches for MCMV and new strategies are under development for improved diagnosis (Zhang et al., 2011).

The genome of MCMV is positive-sense RNA of size 4.4 kb and complete virion is icosahedral with 30 nm diameter (Lommel et al., 1991; Stenger and French, 2008). Genome lacks both the 5′genome linked protein, VPG (viral protein genome-linked) and the 3′polyA tail, and encodes six overlapping open reading frames (ORFs) thereby generates six proteins of distinct molecular weights (Nutter et al., 1989). Mapping from the 5′end of RNA genome, the sequence encodes for P32 (32 kDa protein), RNA dependent RNA polymerases (replicases P50 and P111), P31 (31 kDa protein), P7 (7 kDa protein), coat protein (25 kDa). Two large RNA molecules called sub-genomic RNA1 (sgRNA1) and sub-genomic RNA2 (sgRNA2) are at the 3′end of the genome. sgRNA1 possesses genetic code for expressing P7, P31, coat protein, and also sgRNA2 (Stenger and French, 2008; Deng et al., 2015).

MicroRNAs (miRNAs) are small endogenous ssRNA molecules, 21–23 nucleotides in length, which are formed after processing of hair-pin loop like miRNA precursors (pre-miRNA) by RNase-III like enzyme (Dicer) and can negatively regulate the gene expression (Brodersen and Voinnet, 2006). RNA silencing, through miRNAs present in host plant species, thus imparts natural immunity and resistance to the host against foreign genetic elements including plant viruses (Qu et al., 2007; Iqbal et al., 2016). In maize genome, 321 mature miRNAs have been found (Griffiths-Jones et al., 2008; Kozomara and Griffiths-Jones, 2014); a subset of these mature miRNAs in *Zea mays* should have targets in MCMV genome and these miRNAs, once identified, can be expressed through cloning to enhance the resistance against infection from MCMV (Ali et al., 2014, 2016).

The purpose of this research work was to implement the computational methods for the identification of the targets of Maize-derived microRNAs in the genome of MCMV, as a precedent for enhancing the resistance of the maize plants to MCMV through RNAi. For this objective, a set of miRNAs were retrieved from miRNA databases and were tested against MCMV genome through *in-silico* experiments involving four different miRNA prediction algorithms. The predicted miRNAs can lead to the development of MCMV resistant *Zea mays* plants by using transformation techniques.

## Materials and methods

### Retrieval of mature miRNAs of *zea mays*

The microRNA database, miRBase (Griffiths-Jones et al., 2008; Kozomara and Griffiths-Jones, 2014) accessed from http://www.mirbase.org/cgi-bin/browse.pl, was used to retrieve 321 miRNAs of *Zea mays*.

### MCMV genome retrieval and annotation

**Figure 1:**
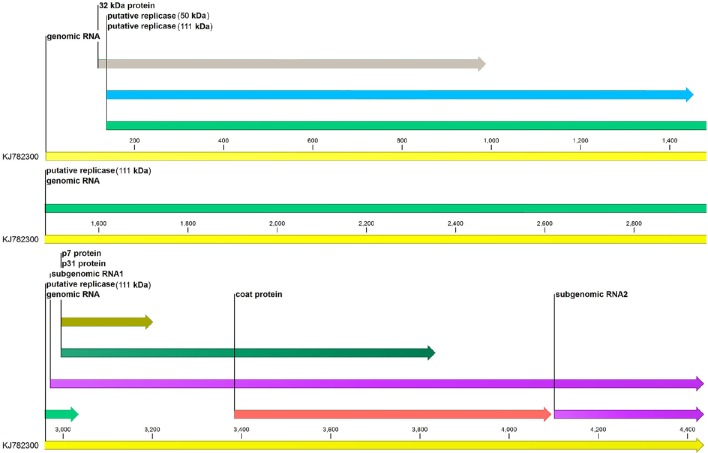
**Genome annotation of maize chlorotic mottle virus**. Six genes (p32, p50, p111, p7, p31, and coat protein) of MCMV are represented along with their sizes. sgRNA1 and sgRNA2 at the 3′end of the genome are also shown.

The complete genome sequence of MCMV with the accession number KJ782300 was downloaded from NCBI and CLC Genomics Workbench (v 9.5.2) was used for annotation of genome features of MCMV (Figure 1).

### miRNA target prediction in MCMV genome

**Figure 2:**
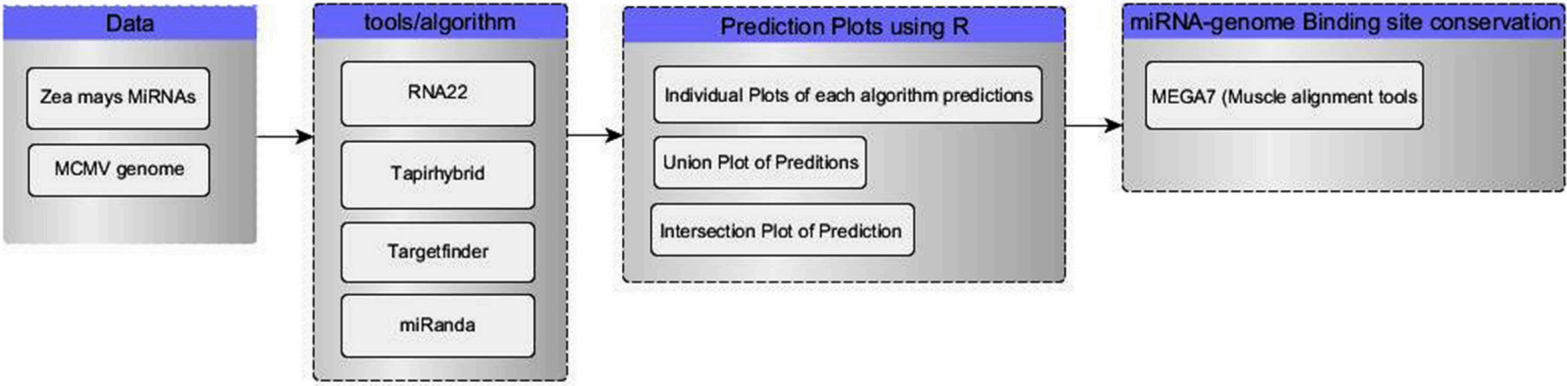
**Flow chart of our miRNA target prediction in MCMV genome pipeline**. Data group contains the type of data acquired for this study from miRBase(miRNAs) and NCBI(virus genome). Algorithm group enlists all the miRNA target prediction tools used in this study. R language was used to make plots and select/refine data using in-house scripts/codes. In the last, binding sites of miRNA-genome attachment conservation throughout other known MCMV strains, were studied using MSA.

Four tools (Table 1) named miRanda, Tapirhybrid, Target finder and RNA22 were used for screening miRNAs of *Zea mays* against MCMV genome to locate miRNA targeting regions. The sequences of Maize miRNAs and the genome of MCMV, both in FASTA format, were processed through these algorithms using the desired parameters. Figure 2 shows a detailed workflow pipeline adopted to predict the miRNA sites within the MCMV genome.

**Table 1:** **Main parameters considered by some of the common miRNA target prediction algorithms**.

**Software/Algorithm**	**Main parameters**	**References**
miRanda	Seed pairing, Target site accessibility, Multiple target sites	John et al., 2004
RNAhybrid	Seed pairing, Target site accessibility, Multiple target sites	Rehmsmeier et al., 2004
Targetfinder	Seed pairing	Fahlgren et al., 2007
Tapirhybrid	Seed pairing, Target site accessibility, Multiple target sites	Bonnet et al., 2010
TargetScan	Seed pairing, Multiple target sites	Lewis et al., 2005
Target-align	Multiple target sites	Xie and Zhang, 2010
Target_Prediction	Target site accessibility	Sun et al., 2011
RNA22	Site complementarity, Pattern recognition and folding energy	Miranda et al., 2006

### miRanda

miRanda algorithm (John et al., 2004) has been used for plant miRNA target prediction (Archak and Nagaraju, 2007; Iqbal et al., 2016). It considers the properties of sequence complementarity, free energy of RNA-RNA duplex and cross-species conservation of the target site to produce the output which is a weighted sum of match and mismatch scores for base pairs and gap penalties. It also promotes the prediction of multiple miRNA target sites including the ones with imperfect binding in the seed region within the 3′UTR of the target site thereby enhancing the specificity (Betel et al., 2008; Witkos et al., 2011). The algorithm was run after defining the settings (gap open penalty = −9.0, gap extend penalty = −4.0, score threshold = 140, energy threshold = −20 kcal/mol and scaling parameter = 4.0).

### Tapirhybrid

Tapirhybrid (Bonnet et al., 2010) considers the parameters of seed pairing, target site accessibility, multiple target sites and is a highly recommended tool for plant miRNA prediction, as it predicts with a greater accuracy as compared to many other tools (Srivastava et al., 2014). Precise mode of algorithm was run which is based on RNAhybrid algorithm, for miRNA target prediction. It generates miRNA target scores after taking into account the number of mismatches, gaps, the number of GU pairs and mismatches in the seed region. Thus, the usage of this tool was primarily to implement a high precision algorithm to enhance the accuracy of the results. The input parameters were set to score <= 8 and mfe_ratio >= 0.5.

### Targetfinder

Targetfinder (Allen et al., 2005; Fahlgren and Carrington, 2010) has been evaluated to be a highly sensitive and accurate algorithm for plant miRNA prediction, with a high percentage of true positive results as compared to other algorithms(Srivastava et al., 2014). It uses FASTA searches to find the potential targets, considering the seed matches, and score them after assessment of penalties for mismatches, bulges, gaps and G:U pairs. Score was set to 10, while the other parameters were set at default.

### RNA22

RNA22 (Miranda et al., 2006; Loher and Rigoutsos, 2012) uses an approach divergent from the other miRNA prediction tools; it implements a pattern-based approach and folding energy to locate the possible miRNA target sites without a cross-species conservation filter. Identification of putative miRNA target sites is also possible even without the identity of the targeting miRNA. The algorithm first analyzes the sequences of known mature miRNAs and then on the basis of pattern information in the miRNAs, it predicts the putative target sites, with many aligned patterns and then identifies the miRNAs which are likely to bind to the predicted target sites. RNA22 was accessed from the web (cm.jefferson.edu/rna22/Interactive/) and the miRNA and the target genome was input to the algorithm. Sensitivity and specificity values were kept at 63% and 61%, respectively and seed size of 7 was selected with 1 unpaired base allowed in the seed region and no limit was set to the maximum number of G:U wobbles in the seed region. Minimum number of paired-up bases was kept to 12 while the maximum folding energy was kept at −14 kcal/mol.

### Statistical analysis

miRNA prediction data obtained from all the four algorithms (Supplementary Table 1) was analyzed by the using R language through RStudio (an integrated development environment for R) (Gandrud, 2013). In-house scripts, Packages readxl and ggplot2 were used in the processing and graphical representation of the result data.

### miRNA-genome binding site conservation analysis

All the MCMV complete genome sequences were retrieved from NCBI nucleotide database available till datebearing accession numbers KJ782300.1, GU138674.1, EU358605.1, JQ982468.1, KF010583.1, JQ982470.1, JQ982469.1, KP851970.3, NC_003627.1, X14736.2. Sequences were analyzed for the conservation of the attachment sites for bioinformatically screened miRNAs using MEGA7 (Kumar et al., 2016). Muscle sequence alignment algorithm was used to align the MCMV genomes. miRNA binding site sequences (identified by the four miRNA-target prediction tools) were aligned to these already aligned genomes using clustalW algorithm, individually.

## Results and discussion

MLND, occurring due to the infection by MCMV, can have adverse effect on the yield of *Zea mays*. Various reports of maize infection with MCMV have been found from around the world (Jiang et al., 1992; Xie et al., 2011; Deng et al., 2015; Wangai et al., 2016). RNAi based silencing the genome of DNA or RNA viruses is a robust technique that can be implemented to enhance viral resistance in plants (Duan et al., 2012). Niu et al. (2006) has reported the silencing of specific genes of turnip yellow mosaic virus (TYMV) and turnip mosaic virus (TuMV) by generating transgenic *Arabidopsis thaliana*. To silence the gene encoding Pns12 protein of *Rice dwarf virus*, Shimizu et al. (2009) cloned and expressed the RNAi constructs in rice to create transgenic rice plants, resistant to *Rice dwarf virus*. To boost the defense system of *Zea mays* against the RNA genome of MCMV, genome encoded miRNAs of *Zea mays* can be expressed after the potential miRNAs of *Zea mays* targeting MCMV genome are found. Hence the core target of this study was to find the miRNA targets in the genome of MCMV, which can be targeted by specific miRNAs of *Zea mays*.

### miRNA target prediction in genome of MCMV

**Figure 3:**
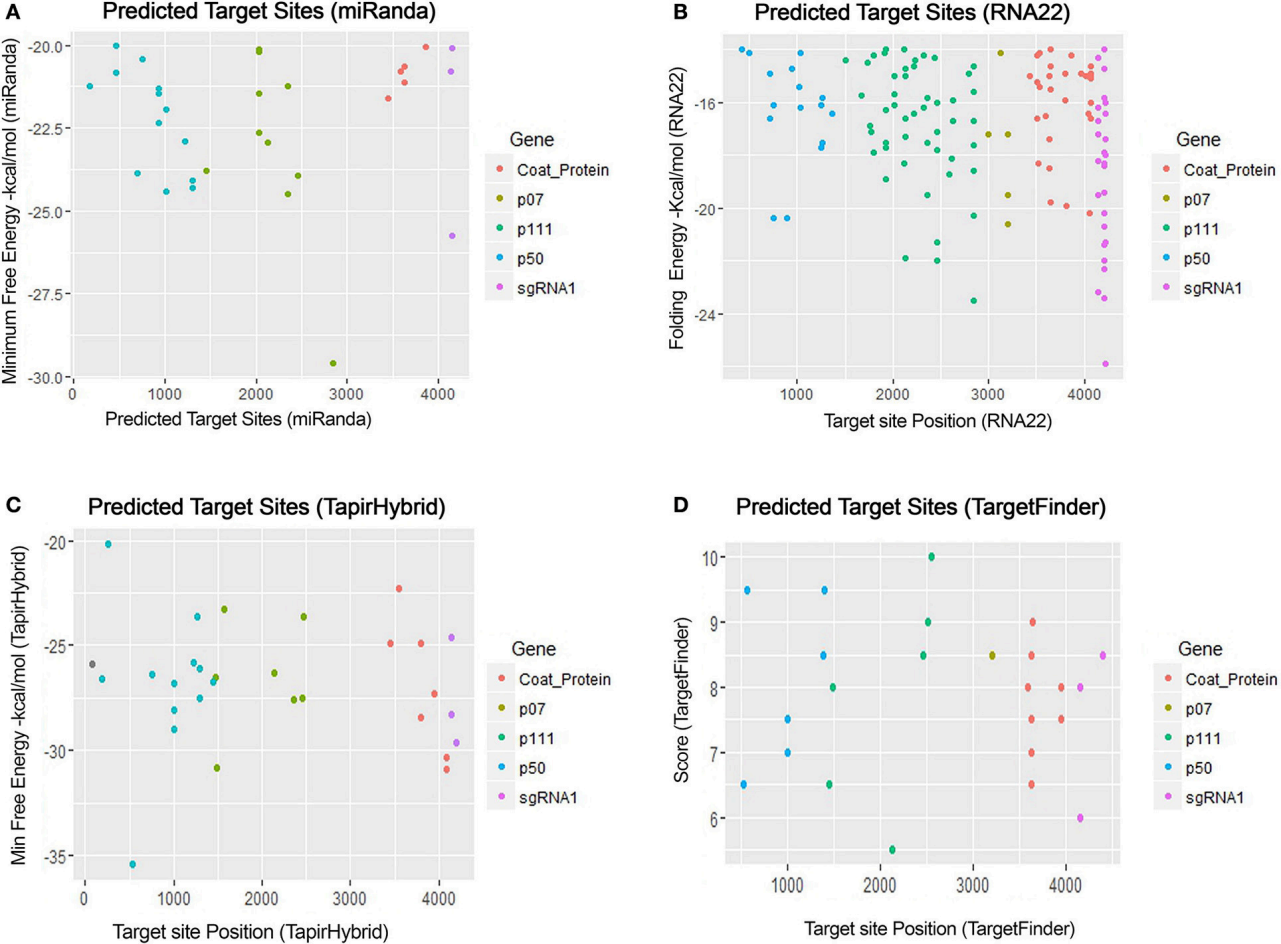
**miRNA Target prediction results of maize chlorotic mottle virus. (A)** Indicates target prediction results from miRanda, **(B)** target prediction by RNA22, **(C)** target prediction by TapirHybrid and **(D)** target prediction by TargetFinder. The color code/key has been linked to the respective figures.

**Figure 4:**
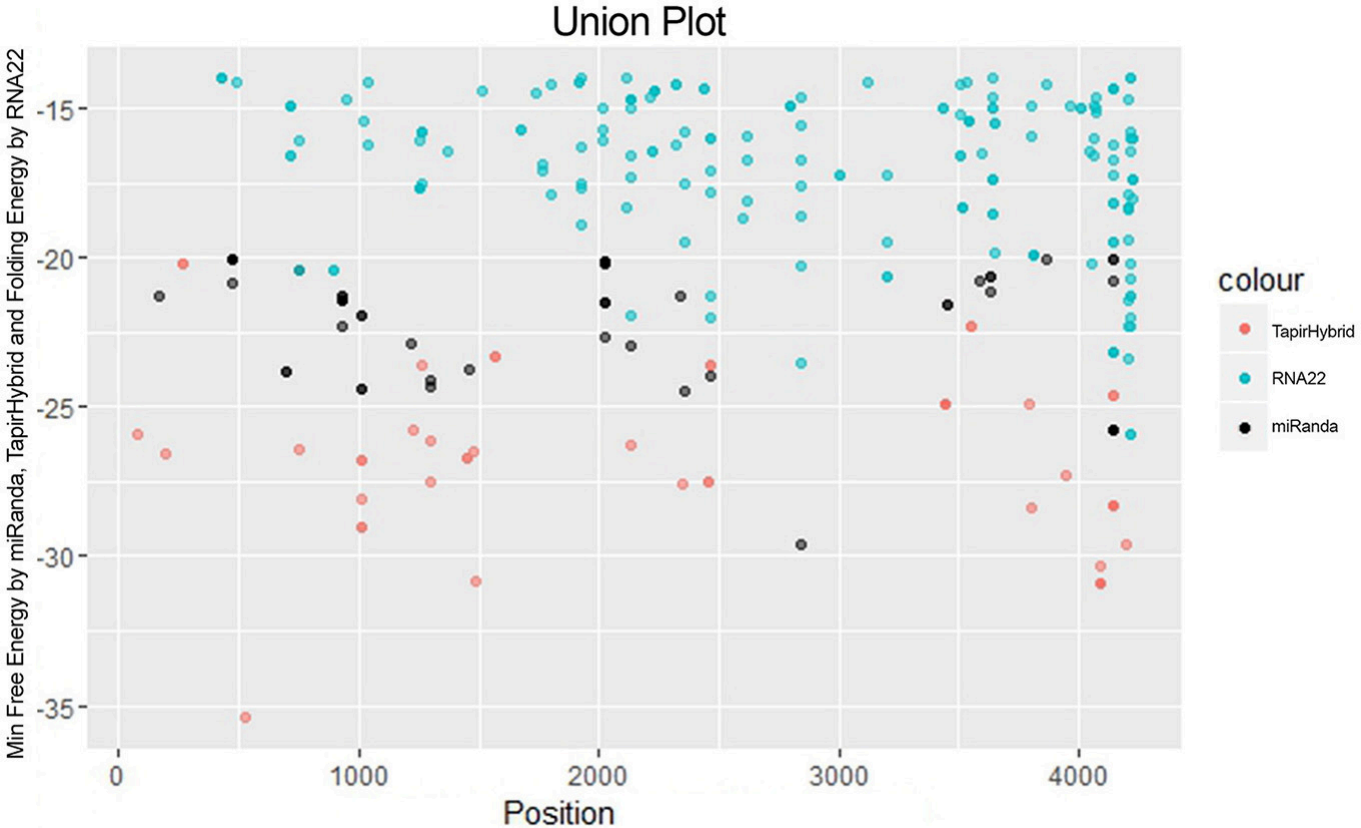
**miRNA Target prediction results shown as a union from three algorithms: TapirHybrid, RNA22, and miRanda**. The color code has been given with the figure.

For prediction of miRNA targets in the genome of MCMV, above mentioned tools were used in combination to maximize the accuracy of miRNA target prediction and for filtering out the false positive results. miRanda (John et al., 2004) was used as it implements various parameters, including target site conservation to predict a large number of miRNA target sites. Afterwards, Targetfinder (Allen et al., 2005; Fahlgren and Carrington, 2010) and Tapirhybrid (Bonnet et al., 2010) were applied which have been recommended for plant miRNA target prediction; various plant miRNA prediction algorithms have been compared by Srivastava et al. (2014) and both of these algorithms have been concluded as the ones giving the most satisfactory output for plant miRNA target prediction. RNA22 (Miranda et al., 2006; Loher and Rigoutsos, 2012) uses an approach that is different from the three algorithms, and predicts on the basis of patter-recognition in the target sequence. Figures 3, 4 shows all the genome positions targeted by *Zea mays* miRNAs by the various algorithms used.

### 32 kDa protein (P32)

A very little is known about the function of this protein. It has been found not be involved in replication and movement but its removal has been found to be associated with decreased level of virus accumulation and disease symptoms (Scheets, 2016).

P32 gene of MCMV was among the least targeted genes by the host derived miRNAs of *Zea mays*. P32 was targeted at two positions by two different miRNAs. Targetfinder and Tapirhybrid predicted binding of miR160a-3p at locus 528 while RNA22 and Tapirhybrid predicted hybridization of miR408b-5p at locus 752.

### P50 and P111 (putative replicases)

P50 and P111 (read-through protein) both contain a GDD box (glycine- aspartate- aspartate motif), also called polymerase domain found in positive strand RNA viruses, and have been predicted to be RNA-dependent RNA replicases (RdRp) as it has been found through comparison with known replicases (Nutter et al., 1989).

For P50 gene of MCMV, 11 targeting miRNAs were predicted (miR160a-3p, miR164c-3p, miR164h-3p, miR166l-5p, miR171b-5p, miR171f-5p, miR319a-5p, miR319c-5p, miR408b-5p, miR528a-3p, and miR528b-3p). The greatest number of potential targets of *Zea mays* miRNAs was for the P111 gene targetted by eighteen different miRNAs (miR156j-3p, miR160a-3p, miR164c-3p, miR164h-3p, miR164h-5p, miR166k-5p, miR166l-5p, miR168b-3p, miR171b-5p, miR171f-5p, miR319a-5p, miR319c-5p, miR399h-5p, miR408b-5p, miR444a, miR444b, miR528a-3p, and miR528b-3p), in combination, at a total of 13 loci of P111 gene. miRNAs targeting both P50 and P111 genes at the same loci were miR164c-3p and miR164h-3p (locus 1009), miR171b-5p and miR171f-5p (locus 1300), miR319a-5p and miR319c-5p (locus 1454), miR444a and miR444b (locus 2466), and miR528a-3p and miR528b-3p (locus 1009).

### P31 (31 kDa protein)

This protein is encoded by a part of subgenomic RNA1 region at the 3′end of MCMV genome and is produced due to suppression of stop codon of the ORF encoding 7 kDa protein (Scheets, 2000). P31 is not involved in cell-to-cell movement; however, it enhances the long distance spread of the virus in plants (Scheets, 2016).

Suitable miRNAs for targeting P31 gene were predicted to be miR169c-3p, miR171b-3p, miR171n-3p, miR395a-5p, and miR399c-5p. Moreover, miR171b-3p, and miR171n-3p targeted the P31 gene at a common locus, while the rest of miRNAs (miR169c-3p, miR395a-5p, and miR399c-5p) hybridized at unique positions in P31 gene.

### P7 (7 kDa protein)

P7 includes proteins P7a and P7b which are similar to the movement protein MP1 and MP2, respectively, found in tombusviridae genera, and are required for cell-to-cell movement of virus in plants (Turina et al., 2000; Yuan et al., 2006; Scheets, 2016). Constructs encoding aberrant transcripts of P7 eliminates the disease symptoms of MCMV in *Zea mays* (Scheets, 2016).

P7 gene had the least number of predicted targets by *Zea mays* miRNAs; only one miRNA of *Zea mays* (miR395a-5p) was targeted at this this gene at locus 3201, indicated by RNA22 and Targetfinder.

### Coat protein (25 kDa)

Full length coat protein is needed for cell-to-cell movement of MCMV virions in plants. Reducing or stopping coat protein production has the effect of impairment of virus infection *in vivo* (Scheets, 2016). Four *Zea mays* miRNAs (miR169c-3p, miR171b-3p, miR171n-3p, and miR399c-5p) were found to have binding sites in coat protein gene at three different loci.

Potential miRNAs for targeting coat protein gene were predicted to be miR169c-3p, miR171b-3p, miR171n-3p, and miR399c-5p. Moreover, miR171b-3p and miR171n-3p targeted the coat protein gene at the same locus, while the rest of predicted miRNAs (miR169c-3p and miR399c-5p) had targets at different positions in coat protein gene.

### Subgenomic RNA1 and RNA2

Subgenomic RNA1 (sgRNA1) expresses all the ORFs in the 3′end of the genome (Scheets, 2000). The proteins encoded by sgRNA1 are P31, P7 (both of which start from the same AUG) and the coat protein (which begins at the second AUG of sgRNA1). sgRNA1 was potentially targeted by 15 miRNAs: miR156i-3p, miR159a-3p, miR159b-3p, miR159c-3p, miR159d-3p, miR159f-3p, miR159h-3p, miR159i-3p, miR159j-3p, miR159k-3p, miR169c-3p, miR171b-3p, miR171n-3p, miR395a-5p, and miR399c-5p.

Subgenomic RNA2 (sgRNA2) is 337 nucleotide a non-coding RNA which accumulates in protoplasts and plants infected with MCMV (Scheets, 2000). However, it is not yet clear whether it is a true sgRNA or a structure-protected degradation product (Iwakawa et al., 2008).

Total 10 potential miRNAs for silencing sgRNA2 were predicted: miR156i-3p, miR159a-3p, miR159b-3p, miR159c-3p, miR159d-3p, miR159f-3p, miR159h-3p, miR159i-3p, miR159j-3p, and miR159k-3p. Since the transcript of sgRNA2 overlaps with the 3′terminal sequence of sgRNA1, miRNA targeting sgRNA2 also hybridized to sgRNA1 at the same genome locus. Those miRNAs targeting sgRNA1 (and not sgRNA2) were exactly the same as those predicted for the P31 gene (i.e., miR169c-3p, miR171b-3p, miR171n-3p, miR395a-5p, and miR399c-5p).

### Maize miRNAs (detected by a consensus of algorithms) for silencing MCMV genome

**Figure 5:**
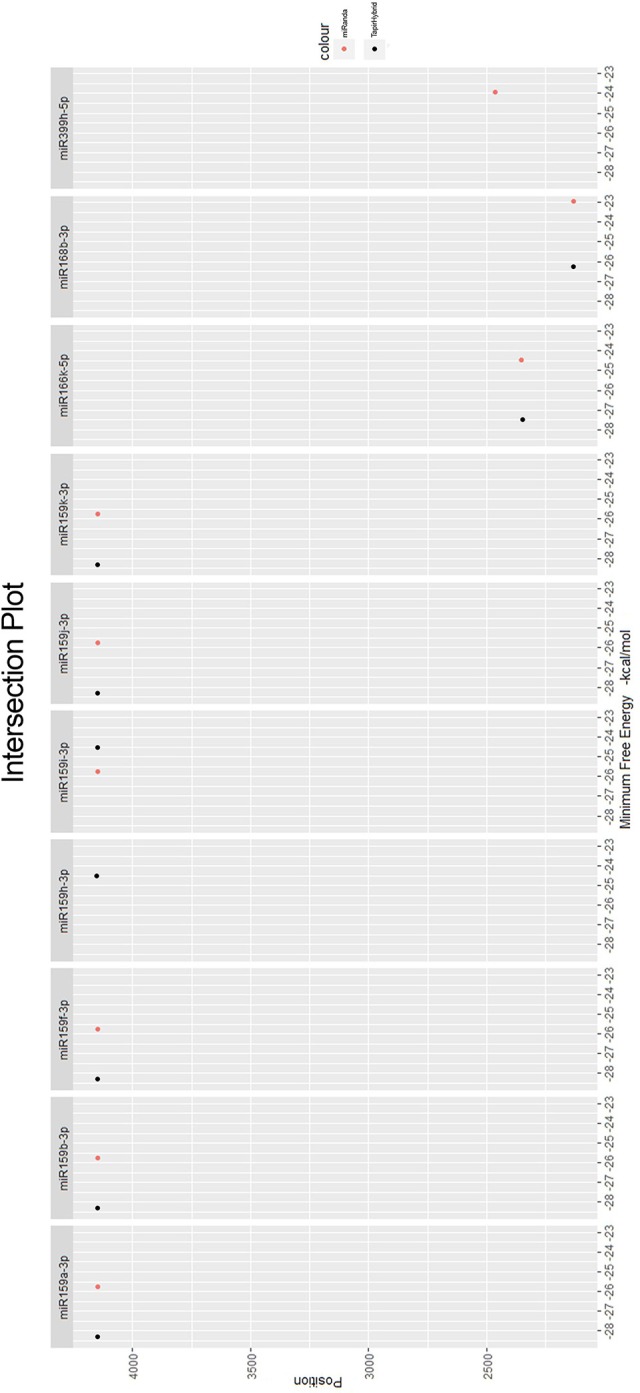
**miRNAs predicted from at least three algorithms**. Minimum free energy (from miRanda and TapirHybrid) has been shown. The color code has been given with the figure.

**Figure 6:**
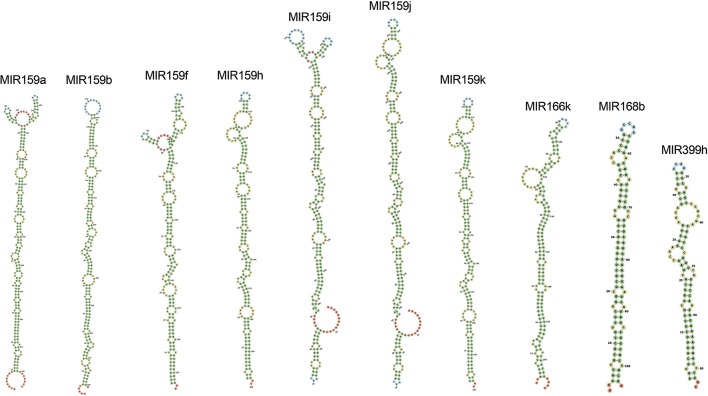
**Secondary structures of pre-miRNAs, precursors of the mature miRNAs found in the study as the miRNAs detected by a consensus of algorithms**. Stems (canonical helices) = green, multiloops (junctions) = red, interior loops = yellow, hairpin loops = blue and 5′ and 3′ unpaired regions = orange.

Among all the targeting miRNAs of *Zea mays* for silencing MCMV genome, ten miRNAs (miR159a-3p, miR159b-3p, miR159f-3p, miR159h-3p, miR159i-3p, miR159j-3p, miR159k-3p, miR166k-5p, miR168b-3p, and miR399h-5p) were predicted by at least three algorithms (including RNA22) or by all the four algorithms used (Figure 5). These miRNAs were classified as the most suitable *Zea mays* miRNAs against the genome of MCMV, since these were predicted after considering the parameters of seed pairing, minimum free energy, target site accessibility, pattern recognition and folding energy thus integrating all aspects of miRNA target prediction and therefore are the most suitable selections for gene silencing. Secondary structures of miRNA precursors have been shown in Figure 6.

### miRNA-genome binding site conservation analysis

**Figure 7:**
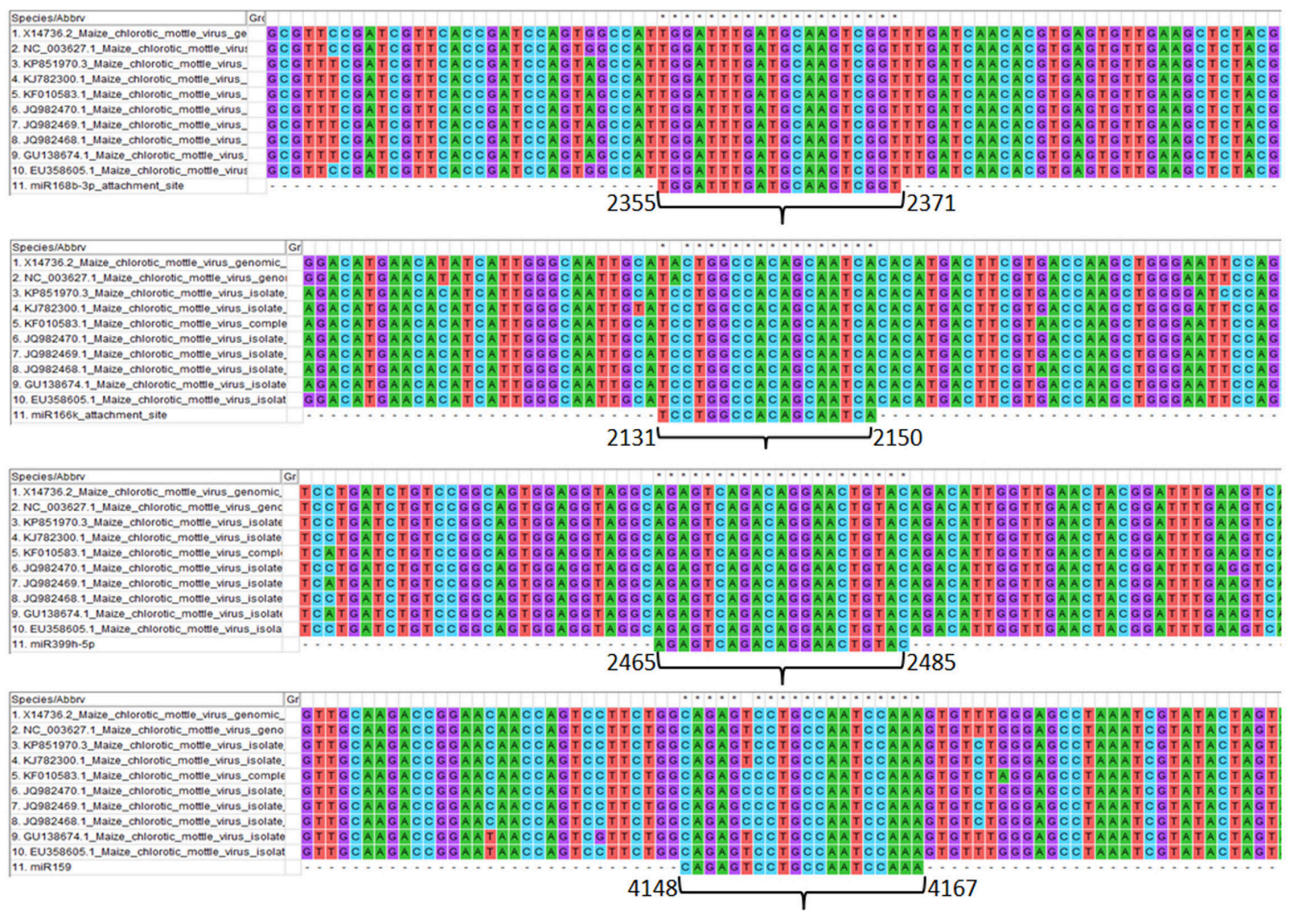
**Multiple sequence alignment of MCMV genomes (available to date at NCBI nucleotide database) showing the conservation of attachment site of the respective miRNAs (sites are numbered according to the KJ782300.1)**. Further, base-by-base nucleotide attachment of miRNAs with the complementary MCMV genome sites can be found in the Supplementary File 1.

Genomic sites serving as an attachment to the respective screened miRNAs show the details of the level of conservation in binding sites in different MCMV strains (Figure 7). This study helps to refine the miRNA selection by investigating the highest binding site conservation level. Furthermore, base-by-base each miRNAs attachment to the complementary MCMV genome sites can be found in the Supplementary File 1.

## Conclusion

This study presents an organized *in silico* approach for finding host-derived miRNAs aimed at silencing the genome of virus affecting the host plant by RNA interference. Through the use of four different algorithms, putative miRNAs have been predicted from the repertoire of *Zea mays* encoded miRNAs. Also, through the MSA study, the level of conserved binding sites of MCMV strains is identified. The short-listed miRNAs, identified as the putative miRNAs of *Zea mays* are the best selections to be used in transformation for the production of MCMV resistant Maize varieties.

## Author contributions

The main idea was developed by MI and bioinformatics analysis were done by BJ and MI. Data analysis was done by MI. The author QA contributed in interpretation of data for the work and English editing. Manuscript was written by BJ and MI. Manuscript was proof-read jointly by all authors.

### Conflict of interest statement

The authors declare that the research was conducted in the absence of any commercial or financial relationships that could be construed as a potential conflict of interest.

## Supplementary material

Supplementary Table 1 indicates the targeting miRNAs predicted from all four algorithms. Genome positions and the output parameters including minimum free energy, folding energy and scores have been included. Supplementary File 1 has the miRNA-MCMV site attachment details containing base-by-base information.
