# Supplementary material for: In silico MCMV Silencing Concludes Potential Host-Derived miRNAs in Maize
Source: Front Plant Sci. 2017 Mar 28;8:372. doi: 10.3389/fpls.2017.00372 (PMC5368279; doi:10.3389/fpls.2017.00372)
Supplement: Supplementary file 2 [file DataSheet1.DOCX]

**miR159 family is divided into 2 groups on the basis of the minor miRNA-genome hybridization variability (shown in Red & blue)**

**>zma-miR159a-3p**

CAGAG-TCCTGCCAATCCAAA MCMV genome
||||| |||: |||||||||
GTCTCGAGGGAAGTTAGGTTT miRNA

Genome position: 4148-4167

**>zma-miR159b-3p**

CAGAG-TCCTGCCAATCCAAA MCMV genome
||||| |||: |||||||||
GTCTCGAGGGAAGTTAGGTTT miRNA

Genome position: 4148-4167

**>zma-miR159f-3p**

CAGAG-TCCTGCCAATCCAAA MCMV genome
||||| |||: |||||||||
GTCTCGAGGGAAGTTAGGTTT miRNA

Genome position: 4148-4167

**>zma-miR159h-3p**

CAGAG-TCCTGCCAATCCAAA MCMV genome
||||| |||: || ||||||
GTCTCGAGGGAAGTGAGGTTT miRNA

Genome position: 4148-4167

**>zma-miR159i-3p**

CAGAG-TCCTGCCAATCCAAA MCMV genome
||||| |||: || ||||||
GTCTCGAGGGAAGTGAGGTTT miRNA

Genome position: 4148-4167

**>zma-miR159j-3p**

CAGAG-TCCTGCCAATCCAAA MCMV genome
||||| |||: |||||||||
GTCTCGAGGGAAGTTAGGTTT miRNA

Genome position: 4148-4167

**>zma-miR159k-3p**

CAGAG-TCCTGCCAATCCAAA MCMV genome
||||| |||: |||||||||
GTCTCGAGGGAAGTTAGGTTT miRNA

Genome position: 4148-4167

**>zma-miR166k-5p**

TCCTG-GCC--ACAGCAATCA MCMV genome
 ||:| ||| |||:|||||
GGGGCTCGGTCTGTTGTTAGG miRNA

Genome position: 2355-2371

**>zma-miR168b-3p**

TGGATTTGATGCAAGTCGGT MCMV genome
 |:|||||||||| |||
AAGTGAACTACGTTCCGCCC miRNA

Genome position: 2131-2150

**>zma-miR399h-5p**

AGAGTCAGACAGGAACTGTAC MCMV genome
 |:|||| :||||||:||
GCACGGTCTCCTCTTGACGTG miRNA

Genome position: 2465-2485
